# Comparative Study Regarding the Properties of Methylene Blue and Proflavine and Their Optimal Concentrations for In Vitro and In Vivo Applications

**DOI:** 10.3390/diagnostics10040223

**Published:** 2020-04-15

**Authors:** Maria-Eliza Nedu, Mihaela Tertis, Cecilia Cristea, Alexandru Valentin Georgescu

**Affiliations:** 1 Department of Plastic Surgery, Faculty of Medicine, Iuliu Hațieganu University of Medicine and Pharmacy, 46-50 Viilor St., 400347 Cluj-Napoca, Romania; eliza.nedu@gmail.com (M.-E.N.); valentine.georgescu@umfcluj.ro (A.V.G.); 2 Department of Analytical Chemistry, Faculty of Pharmacy, Iuliu Hațieganu University of Medicine and Pharmacy, 4 Pasteur St., 400349 Cluj-Napoca, Romania; mihaela.tertis@umfcluj.ro

**Keywords:** methylene blue, proflavine, phenothiazine, acridine, dye, antibacterial, surgery

## Abstract

Methylene blue and proflavine are fluorescent dyes used to stain nucleic acid from the molecular level to the tissue level. Already clinically used for sentinel node mapping, detection of neuroendocrine tumors, methemoglobinemia, septic shock, ifosfamide-induced encephalopathy, and photodynamic inactivation of RNA viruses, the antimicrobial, anti-inflammatory, and antioxidant effect of methylene blue has been demonstrated in different in vitro and in vivo studies. Proflavine was used as a disinfectant and bacteriostatic agent against many gram-positive bacteria, as well as a urinary antiseptic involved in highlighting cell nuclei. At the tissue level, the anti-inflammatory effects of methylene blue protect against pulmonary, renal, cardiac, pancreatic, ischemic-reperfusion lesions, and fevers. First used for their antiseptic and antiviral activity, respectively, methylene blue and proflavine turned out to be excellent dyes for diagnostic and treatment purposes. In vitro and in vivo studies demonstrated that both dyes are efficient as perfusion and tissue tracers and permitted to evaluate the minimal efficient concentration in different species, as well as their pharmacokinetics and toxicity. This review aims to identify the optimal concentrations of methylene blue and proflavine that can be used for in vivo experiments to highlight the vascularization of the skin in the case of a perforasome (both as a tissue tracer and in vascular mapping), as well as their effects on tissues. This review is intended to be a comparative and critical presentation of the possible applications of methylene blue (MB) and proflavine (PRO) in the surgical field, and the relevant biomedical findings from specialized literature to date are discussed as well.

## 1. Introduction

Methylene blue (MB) is a phenothiazine derivative dye that produces a blue color when dissolved in water. It is used as a nucleic acid stain for its ability to bind DNA and RNA. Due to the fact that MB acts as a cationic dye that forms electrostatic bonds with negatively-charged moieties, it is intensively used as redox probe and intercalator in designing DNA and aptamer-based sensors [1,2,3]. This reversible staining minimizes interference when nucleic acids are transferred to hybridization membranes for blotting [4].

MB administration exhibits complex pharmacokinetics because of multiphasic distribution into various tissue compartments along with a slow terminal rate of disappearance. MB is excreted uncontrolled in the urine at any time between 4 and 24 h after administration, with a biological half-life of 5 to 6.5 h [2].

The small molecular weight of MB allows its easy and fast diffusion into tissue; thus, it is widely used in many intraoperative procedures. The ease of visualizing the dark blue color of MB with unaided eyes during surgery facilitated its use for sentinel node mapping. However, high concentrations of this dye are needed to visualize the blue color. Recent studies have shown that MB presents fluorescence at about 700 nm, thus, it can provide a mechanism for the detection of the distribution of this dye through highly sensitive sensing systems [5]. This dye has also been applied to the detection of neuroendocrine tumors, such as insulinoma [6].

Also known as a biologic dye, proflavine (PRO) is an acriflavine derivative, used as a disinfectant and bacteriostatic agent against many gram-positive bacteria as well as a urinary antiseptic. PRO is also known to have a mutagenic effect on DNA by intercalating between nucleic acid base pairs; this makes PRO a special type of mutagenic compound because it causes base pair-deletions or base pair-insertions rather than substitutions. In the presence of light, PRO can also induce double-stranded breaks in DNA [7,8].

The reason why the use of PRO on human subjects is limited in the United States is that it is considered a dye with high hazardous potential and a mutagenic nature. Given these assumptions, to date, PRO has mainly been used as an investigational drug [9].

**Figure 1:**
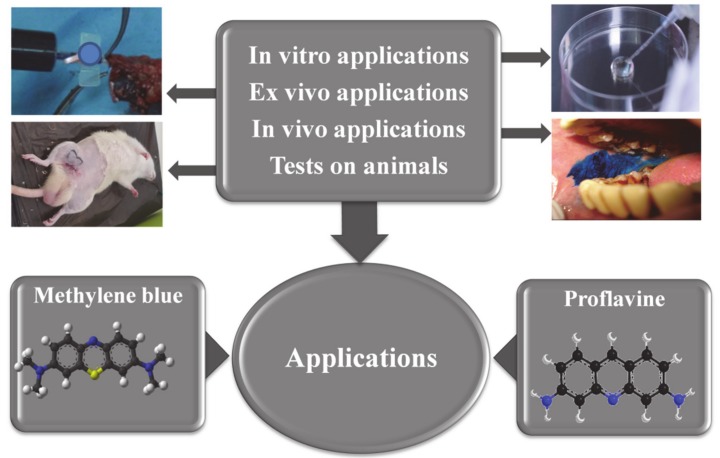
Schematic representation of research directions in biomedical and clinical domains regarding the possible applications of methylene blue (MB) and proflavine (PRO). Chemical structures of MB and PRO are presented at the bottom of the figure.

As hetero-tricyclic dyes (Figure 1), both substances have florescent proprieties which are used in many clinical and experimental applications. When MB, a promethazine drug, is intended for use in in vivo tests, its ability to form bonds with blood cells and easily penetrate inside the cell is taken in consideration [10]. This is supported by its concentration levels in blood that have been found to be four to five times higher than in plasma [11]. Among their clinical and experimental applications [4,12,13,14,15], the most common directions of research related with the use of MB and PRO in biomedical and clinical fields are schematically presented in Figure 1.

The recommended doses for the clinical applications of MB are already well known and are between 1–2 mg/kg/day, while signs of toxicity, like the formation of Heinz bodies in human erythrocytes, appear at much higher concentrations, namely 7.5 mg/kg/day. Doses between 1–2 mg/kg/day are currently used for systemic treatments, but higher doses of 2.5 mg/kg/day could also be used, for example, in some chronic cases. This was demonstrated in a specific experiment performed with mice where this compound was used as medicine in order to extend the life span of the animal [16].

Even though it has not yet been experimentally tested, a theoretical study supported the safe systemic use of MB in the case of pregnant women. This study showed that due to the dilution level there should be no side effects on the fetus [17].

There are some clinical applications approved by the Food and Drug Administration (FDA) for MB among which are the treatments of methemoglobinemia, where in inhered methemoglobinemia daily doses of 50–250 mg are used for a lifetime; in acute methemoglobinemia dosages of 1.3 mg/kg are used once or twice per day with intravenous (i.v.) administration over 20 min [18]; in septic shock, 200 mg are i.v. administrated over 1 h, followed by infusion of 0.25–2 mg/kg/h; and for ifosfamide-induced encephalopathy, doses of 4 × 50 mg/day per os (p.o.) are used [18,19]. An interesting and recent review article centralized several protocols for administrating MB in the treatment of malaria to date. Thus, in the 19^th^ century, boluses of 300–1000 mg per day, divided into five doses were administrated in adults, while only 20–300 mg per day in five doses were administrated in children. This treatment was administrated for 3–90 days, as daily or interrupted (e.g., a two-day pause after several days of treatment) oral monotherapy, with largely varying follow-up periods. In a more recent study, the administration of MB to adults at a unique dose of 500 mg per day for 14 days in combination with isopentaquine (IQ) or in combination with IQ and quinine (Q) was reported [20,21]. Another strategy is to administrate MB to children in various combination regimens (MB-amodiaquine, MB-artesunate) in doses ranging from 4 mg/kg per day to 24 mg/kg per day, initially as 2–4 divided doses per day, but no more than 3 days [22,23]. An additional strategy is to gradually replace MB with new synthetic antimalarials with different characteristics and finally without coloring properties; some are synthesized based on MB itself, as was the case for the first synthetic drug designed as an antimalarial, pamaquine [24].

MB has other applications due to its antimicrobial, anti-inflammatory, and antioxidant effects [10]. In the last decade it has been successfully used in several pathologies [5], as mentioned in Table 1.

MB is the first synthetic dye ever used as an antiseptic in clinical therapy [11]. This effect is granted by its capacity to stain the nucleic acids; moreover, MB is a photosensitizing agent for photodynamic inactivation of RNA viruses including human immunodeficiency virus (HIV), hepatitis B virus, and hepatitis C virus in plasma, while oxidative damage to isolated DNA caused by exposure to UV light is minimal in humans [34].

Similar to MB, PRO has antibacterial effects as reported by Wallis and Melnickr. These authors demonstrated that certain heterotricyclic dyes could be bound irreversibly to the herpes virus. Special attention was given to PRO, mainly due to its ability to penetrate in the nuclei of the cells of the epidermis, thus being used as a topical antibacterial agent. The basis of its antibacterial property is its action as a nucleic acid intercalator. As a result of this important property, PRO is routinely used for umbilical cord care in newborn infants in the United States, with rare episodes of toxicity reported thus far [35].

Both these dyes have been used against gram-positive bacteria in wound dressings [11]. The effects that MB has on tissues derive from its high solubility in water, chloroform, and sparingly in alcohol. MB was used for the first time by Ehrlich, in 1886, to stain the nervous tissue by injecting it intravenously into a living animal [11]. As a non-toxic biological stain, with an affinity for mucosal tissue [9], nowadays, MB is used for nervous tissue and endocrine gland identification during surgery [19]. Meanwhile, PRO is used to highlight the cell nuclei and enable direct visualization of cellular morphology without removing the tissue, on behalf of its property as a topical contrast agent [36].

Different authors reported the use of both dyes for diagnostic procedures including: in vivo confocal laser endomicroscopy [37,38], in vivo microscopy to identify Barrett’s esophagus [39], pathological changes in oral mucosa [40], colon [9], stomach [41], duodenum, upper gastrointestinal tract [41] and central airway, cervical tissue [35,42], cervix [35], sarcoma [43], urinary fistula [44], sentinel node (SN)’s, head and neck cancers [45], breast cancer [17], and gastrointestinal and thyroid surgery [10]. Apart from MB and PRO’s ability to stain nuclei, the latter also stains non-specifically the membranes of adipocyte cells, making it possible to identify adipocytes without using a contrast agent [43]. It is used to distinguish between benign and neoplastic mucosa in the head and neck although hyperspectral imaging proved to be superior. In addition to the fact that fluorescence-guided imaging is used to guide surgical resection in cancer [46], it is also responsible for the increased number of complete resections and progression-free survival [45,47].

Numerous studies have reported the possible therapeutic effect of MB in Alzheimer’s disease treatment. This effect has been attributed to the special anti-inflammatory property of MB [11]. Some particular studies suggested that doses of 60 mg of MB administered three times per day seemed to be efficient for this therapeutic purpose; yet, additional data obtained from clinical trials are needed to allow the use of this dye in therapy schemes for neurological disorders [18,48]. MB is used also for prevention of urinary tract infection in elderly patients, with a dosage of 3 × 65 mg per day applied in this case [19].

The above-mentioned applications have clear dose indication due to the complications that can occur during treatment. Despite the well-known properties of MB, new features are still investigated in detail and other clinical uses are under study without having established the right dose yet. The therapeutic effect is influenced by the concentration of the drug and site of administration. This is the reason why the latest approaches regarding MB and PRO implications in clinical uses are summarized and critically discussed in this review.

Taking into consideration the safety in clinical utilization of both dyes, this paper also aims to review the in vitro and in vivo effects of MB and PRO on concentration and administration routes in order to determine which of the two dyes has the lowest concentration that could be used as a vascular mapping tracer and its effects on tissues and cells.

## 2. Biological Actions of Methylene Blue and Proflavine and Methods

Reviewing the action of both dyes at the biological (cellular) level, not only permits the evaluation of side effects but also the assets of using them apart from their main purpose as a tissue tracer.

### 2.1. Methylene Blue

Any interference with a tissue, like the introduction of a tissue tracer, will lead to inflammation. Proven by many studies, the anti-inflammatory action of MB protects against lesions such as pulmonary lesions induced by endotoxins, bacterial lipopolysaccharides that cause fevers, renal lesions made by ciclosporin, cardiac lesions given by the use of doxorubicin, pancreatic lesions made by streptozotocin, ischemic-reperfusion lesions, and lipid peroxidation suppression and increased inflammation by the time of reperfusion. It also accentuates the long-chain fatty acids oxidation [11,16].

Another important biological feature for the eventual use of the dye as a tissue tracer is its low molecular weight. MB is a partially liposoluble agent, which makes it a rapidly penetrating histological stain [49] that passes easily to the cellular membrane, accumulates in the mitochondrial matrix, enhances respiration which is realized via different processes (cytochrome C oxidase activity, O_2_ consumption, ATP production), minimizes electrons leakage at the level of electron transport chain, and also decreases superoxide formation by the time of reperfusion [11,46].

At the cellular level MB modulates the function of different integral membrane proteins involved in solute transport like glucose and cations, such as Na^+^, K^+^, and H^+^. In addition, MB influences the function of voltage sensitive ions Na^+^, Ca^2+^, Ca^2+^ activated, and K^+^ channels, altering the excitability of neurons and making it suitable for use as a local anesthetic [11,50,51,52]. There are several factors that influence the molecular mechanisms which mediate MB effects, like routes of administration/application, light exposure, membrane potential, or the redox state of the cells [11].

**Figure 2:**
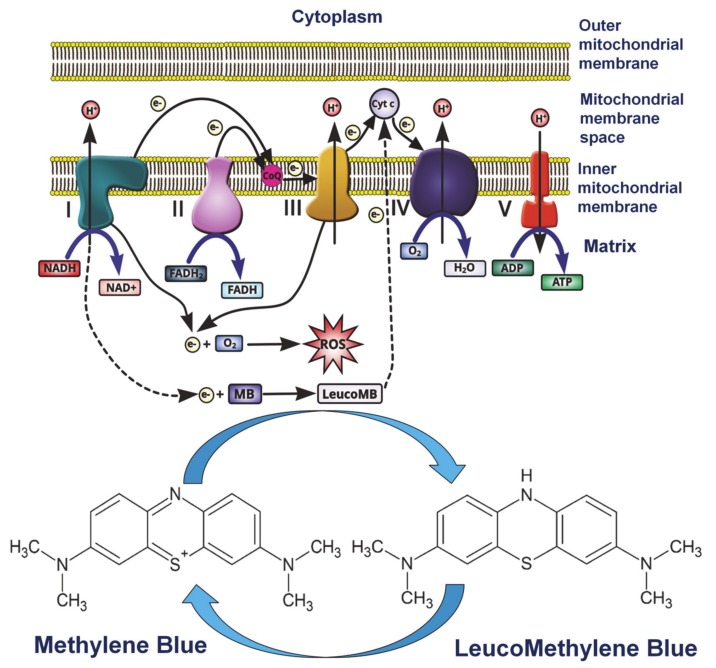
Diagram of MB as an alternative mitochondrial electron transporter. More details can be found in text. Reproduced with permission from [53].

When applied topically, MB improved fibroblast proliferation as well as collagen and elastin fibers production in tegument [34]. At the mitochondrial level, MB acts on the first, third, and fourth respiratory complex. The first complex, facing the cytosol, catalyzes the oxidation of hydrogen nicotinamide adenine dinucleotide (NADH), which gives two electrons to MB. In this case MB acts as an electron carrier, generating the LeucoMB form that is partially formed in the mitochondrial matrix [16]. Since MB has the redox potential of 11 mV, it suffers from the reduction process, thus allowing the continuous cycle between the two forms: MB and LeucoMB, as can be noticed in Figure 2 [53].

The last compound presents redox indicator proprieties, which can be exploited in quantitative analysis of a large number of reductive agents, like glucose and ascorbic acid. Complex IV consumes more than 95% from O2 at the cellular level and the production of H2O2 and other oxidants at the level of complex I or III seems to be improved after blocking complex IV, thus reducing superoxide formation by the time of reperfusion [54]. Furthermore, complex IV assures that cytochrome c recycling is in its reduced form. The MB cycle takes place in mitochondrial electron transport chain (ETC) inhibiting the production of radical superoxide, competing with O_2_ at the place of production of free radicals by NADH dehydrogenase of complex I. Cytochrome c is the electron transporter from complex III to complex IV, with complex I being the natural enzyme which reduces cytochrome c while complex II oxidizes it [53,55].

Decreasing NADH/NAD^+^ rapport, MB prevents cytoplasm acidification, the inhibition of glycogenosis and possibly oxidative metabolism including cyclooxygenase (COX) activity. In complex I and complex III there are two sites which are responsible for free radicals’ production by nonspecific transfer of electrons to O_2_. Despite that one of the cell NAD(P)H-dependent dehydrogenase, the NADH-dehydrogenase of complex I is able to reduce artificial electron acceptors such as MB to MBH2 or O2 to superoxide radical. The cytochrome c, in the mitochondria, and methemoglobin are the only heme proteins reported to reoxidize MBH2 to MB [55]. This interaction between methemoglobin and MB leads to different effects of MB in a concentration-dependent manner as follows: high concentrations convert ferrous iron of reduced hemoglobin (Hmb) to the ferric form methemoglobin. Low concentrations have the opposite effect in drug-induced methemoglobinemia [56].

Furthermore, MB can stabilize the energetic metabolism and increase ATP synthesis after reoxygenation. When the reperfusion takes place the mitochondrial respiratory chain dysfunction leads to reactive oxygen species (ROS) accumulation. ROS affect mitochondrial membranes, targeting lipid peroxidation and mitochondrial lesions which lead to cytochrome c release in cytosol through mitochondrial permeability transition pore (MPTP), activating caspase 3 to induce apoptosis [54].

MB improves mitochondrial function by inducing peroxisome proliferator-activated receptor γ coactivator 1 PGC1α, a central mediator of mitochondrial biogenesis [56], and downregulates the expression of NACHT, Leucine-rich repeat (LRR), and Pyrin (PYD) dominions that contain protein 3 (NLRP3) an inflammasome produced by bone marrow derivatives from macrophages induced by ATP. In addition, MB might regulate inflammation, influencing the signaling way of pro-inflammatory nuclear factor-κB (NF-κB) and inhibiting caspase-1 activation to reduce apoptosis [57].

Post ischemic MB inhibits superoxide production competing with molecular oxygen at the iron-sulfur center of xanthine oxidase, acting as a willing electron acceptor for the last shunting of electron flow from the normal pathway. This switch in favor of an iron-sulfur center allows the anaerobic oxidation of purine substrates, short-circuiting O_2_ generation at the flavin centers. Leucomethylene blue auto-oxidation produces predominantly hydrogen peroxide to the detriment of superoxide. Due to these facts, MB was proposed to attenuate the reperfusion pathophysiology after lesion in two ways. Thus, if MB is administrated before the ischemia, it causes attenuation of the hypoxanthine accumulation and permits anaerobic breakdown of hypoxanthine and xanthine to uric acid. The second option is related to the administration of MB immediately before injury or reperfusion to prevent superoxide formation [49].

All the effects produced by MB on the mitochondria, respiration, and antioxidant effects, are useful tools to reduce the tissue reaction at the invasiveness of a foreign substance on a living tissue. 

### 2.2. Proflavine

In the case of PRO, studies demonstrate that it interacts with erythrocytes at an equilibrium constant of 10^4^ M^−1^ which determines the formation of a complex between PRO and Hgb involved in the transfer of the fluorescence and energy. The energy is transferred from Hgb to the dye which is able to induce relevant secondary changes like the decrease of α helical content and destabilization in Hgb, with this bond being characterized by positive entropy and negative enthalpy [58]. Despite these transformations, essential morphological features of Hgb are retained in the presence of PRO which made it suitable to be used at the intra vascular level [59]. An important issue for intravascular administration purposes is that PRO forms a bond with albumin by hydrogen bonds in aqueous solution with pH 7; under the protonate form, the first one keeps the cationic state by the interaction time with the protein. When the pH increases to 9.5, in the same aqueous solution, a blue shift of the lowest absorption band by 0.36 eV occurs [59].

At the cellular level, PRO binds to potassium channels at the transmembrane domain; it prevents direct Kir 3.2 activity, attenuates Kir 3.2 current in a concentration-dependent manner in depolarized membrane potentials, and inhibits cells which express Kir 3.2 growths. Kir 3.2 is a protein subunit included in proteins that builds protein G channels which internalizes potassium K in the cell. This represents an important feature in treatment of associated neurological disease, including Down’s syndrome. The concentration-dependent manner is explained by multiple ways of forming bonds, although it is a poor blocker. PRO is also an indirect inhibitor of m2-muscarinic receptor (m2R), its effect being observed when Kir3.2/WT is activated by m2. The Kir3.1 and Kir3.2/WT channels are blocked by PRO-WT interactions. The last one explains the selective inhibition of potassium channels. Furthermore, Kir 3.2 may be blocked by hydrophobic acridine nuclei of PRO, which is in the protonated form at neutral pH [60].

As dye, PRO predominantly colors the nuclear material, but in the case of adipocytes, the dye concentrates to non-specifically stained cell membranes, helping in the detection of adipose tissue changes in breast cancer, since it can be observed with the aid of confocal imaging [43].

At the nuclear level, when PRO intercalates DNA, it adopts a minimal base-stacking penalty pathway sustaining the cavity-induced molecular mechanism in a noncovalent manner [16,36].

Like in the case of MB, PRO bounds easily when the phosphate (PO_4_^-^) concentration is higher and also PRO concentration influences the thermodynamic stability of the two states, leading to the interaction between PRO molecules and forming aggregates or micelles on DNA at a concentration of 2.5 μM. The ratio between phosphate (P) and dye (PRO) determines the intercalate complex formation and extern complex that form bonds with DNA as follows: if this ratio is ≤1, the visible absorption spectrum shows a blue shift that is related with the PRO stacking interaction; a ratio of about 10 determines a red shift in the visible absorption spectrum, related with the formation of an intercalated complex; for concentrations below 0.1 nM, the dimers do not form, while for concentrations greater than 10 mM, larger aggregates are formed. An important aspect that has to be taken into consideration when a vehicle is chosen for PRO administration is that sodium chloride (NaCl) molecules associated with Mg ions determine the decrease of the bounding process, thus raising the first compound concentration. This process determines the decrease in the lifetime for the bounding of PRO molecules at the end of polymeric aggregates. For P/PRO ratio of 22, when single PRO and 12 base pairs of DNA are present, there are no external bounds on PRO giving free energetic stability [61].

PRO seems to alter the microenvironment of tryptophan residues compared with tyrosine residues [60]. Another effect of PRO observed at the nuclear level is represented by its antitumoral effect [31] with only minimal damage to DNA, which is then potentiated by immune recruitment and, as a non-covalent binder to DNA, it can act as an indirect cyclic GMP-AMP Synthase cGAS agonist [62].

## 3. In Vitro Studies Using Methylene Blue and Proflavine 

Many in vitro studies were performed in order to determine both the toxic and the most efficient dose of MB and PRO, as well as new effects on different types of tissues.

### 3.1. Methylene Blue Uses for In Vitro Studies

In pharmacological studies, concentrations between 1 and 10 µM of MB were used to inhibit soluble guanylate cyclase and at 5.3 µM it acts as a direct inhibitor of nitric oxide synthases (NOS) on endothelial cells culture; this is important if the intra-arterial administration of the dye is envisaged [63].

At concentrations of 1.1 µM in human plasma, and 0.42 µM in bovine plasma, the dye seems to inhibit the esterase activity that is directly dependent on acetylcholinesterase (AchE) concentration [11,64]. MB concentrations higher than 0.57 μM competitively inhibit AChE activity in cow erythrocytes, but this effect decreases when higher incubation times were used [11]. MB concentrations from 10 nM to 1 µM decrease in iron uptake, thus heme synthesis is decreased by 50% [16].

In the case of intravascular injections, MB can reach the central nervous system, taking into consideration its tropism on the nervous system; this is dependent on the concentration used for the dye. After performing some in vitro tests on cell cultures obtained from an immature rat cerebellum, it was observed that when MB solutions with concentrations between 5 and 50 µM were deposited on rat hippocampal slices, glutamate mediated synapsis was suppressed. In this case, progressive destruction of differentiating cells was observed in the presence of MB. If applied on astrocytes chronically exposed to ethanol, MB protects them by inducing acetaldehyde accumulation and potentiating the in vitro toxic effects of ethanol [11].

MB also seems to protect the brain by reversing accelerated in vitro senescence in Hep G2 cells due to oxidative stress and induces antioxidant defense towards enzymes, thus protecting against irradiation, brain damage, and poisoning in chemotherapy [16,65].

By applying a light source on MB, the energy is absorbed directly and transferred by two mechanisms to either electrons (type I mechanism) or energy (type II mechanism) to molecular oxygen. A singlet oxygen is formed which oxidizes directly electron-rich double bonds in biological molecules and macromolecules. This effect makes the first mechanism suitable as a photosensitizer in cancer treatment and protects serum from viral agents by formatting hydroxyl radicals and lipid hydroperoxides. This process determines the breakage of nucleic acids, and it is especially encountered in the case of guanosine sites, proteins, and lipids. Lipid peroxidation affects membrane integrity, leading to a loss of fluidity and alterations in the functions of several ion channels, receptors, and transporters [10,59].

MB used as a photosensitizer with photodynamic therapy is most effective for in vitro tests. MB mediated photobiomodulation on human osteoblast cells in concentrations between 0.05 and 5.0 µM was studied and a concentration over 0.5 µM seemed to be toxic in this case. A concentration of 0.05 µM MB in combination with a laser was used to study the effect on human osteoblastic cells. Application of light for 72 h seems to visibly affect cell viability. When these types of cells were incubated for 1 h and 7 days respectively at 37 °C with MB, reduced mineralization was observed, this being combined with three energy densities that influence alkaline phosphatase activity [66].

When MB comes into direct contact with the skin, it has an effect on delayed senescence of fibroblasts, this being due to its capacity to change its form from oxidized to reduce as an electron-cycler. It was thus observed that a MB concentration of 100 nM has maximum effect in the presence of 20% O_2_. This phenomenon is due to the interaction of MB with specific mitochondrial electron carriers by giving it respirator enhancing proprieties and increasing oxygen consumption by 70%, thus protecting fibroblasts against oxidative stress [16,63]. MB concentrations of 250, 500, and 1000 μg/mL present slight cytotoxicity. In human embryonic fibroblasts, MB extends proliferative lifespan and increases activity of mitochondrial complex IV as well as mitochondrial hem synthesis in these fibroblasts [65].

Another study evaluated the effect of using MB solutions prepared by dissolving 125 mg of dye powders in 250 mL of distilled water then adding various concentrations of MB on epichlorohydrin epoxidated with a hollow silica sphere (HSS) functionalized with gum Arabic (HSEPCGUM-MB from 10 to 1000 μg/mL) on Swiss 3 T3 albino mouse fibroblast cell line (ATCC-CCL-92) for 72 h. Fibroblasts migration and proliferation into the wounded monolayer was observed after scratch assay, which covers the second phase of wound healing characterized by a proliferation and migration of either keratinocytes or fibroblasts, on cells treated with different dilutions. Adsorption studies of MB used different concentrations ranging from 5 to 50 mg/mL onto 0.01 g adsorbent at 35 °C. At concentrations of 10, 50, and 100 μg/mL no cytotoxicity was observed after 72 h together with a moderate migration activity in a concentration-dependent manner. MB seems to improve scratch closure between 13.23% and 54.81% compared to negative control after 72 h of wounding [67].

MB is also an excellent dye to stain the skin. Thus, by using solution of 0.1% MB prepared in hydroxyethyl cellulose in different liquid crystalline phases, it was observed that it stains strongly fluorescent the stratum corneum and viable epidermis. These observations were obtained after some in vitro studies performed on skin samples taken from pig ear. The fluorescent stain was homogenously distributed and seemed to penetrate not only the skin appendages but also the stratum corneum. The systems with the lowest aqueous content produced hemorrhages and few spots of lysis were milder for a higher aqueous ratio. However, the use of MB aqueous solutions has maximized the dye penetration but with lower efficacy compared with the control gel. This phenomenon should be explained based on the hydration of the corneocyte proteins when in contact with the liquid crystalline phase that may occur at the hydrophilic parts of the protein. This process determines the increase of the interlamellar volume of the lipid bilayer, leading to a disorganization of the stratum corneum barrier that favors the partitioning of hydrophilic drugs like MB [68].

**Figure 3:**
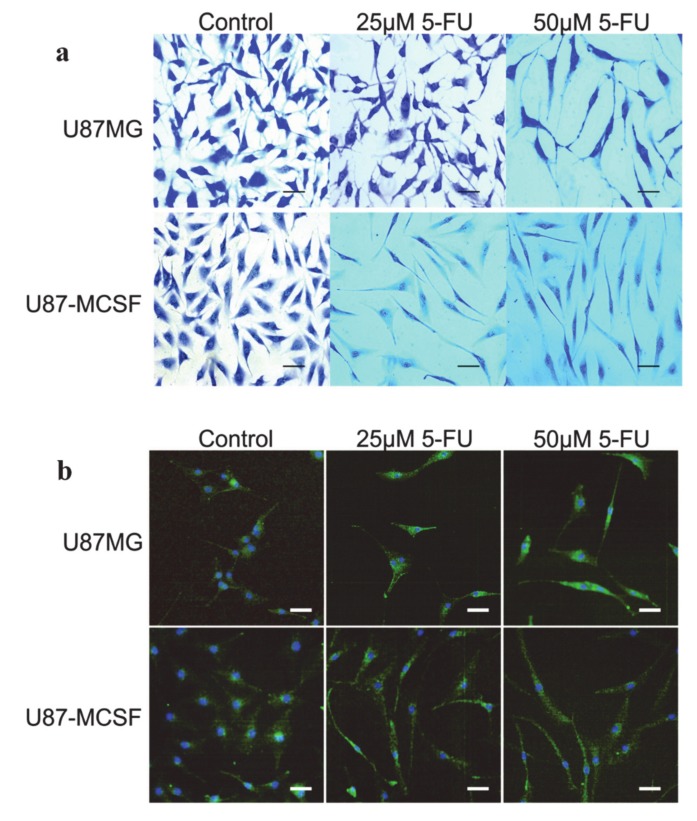
Microscopic examination of morphology of cells after 72 h of 5-fluorouracil (5-FU) treatment. (**a**) Methylene blue staining for detection of cell morphology after 5-FU treatment. (**b**) Actin cytoskeleton staining of treated cells using anti *β*-actin antibody. The morphological pictures show the presence of elongated and mesenchymal cells in U87-MCSF cells treated with 25 µM 5-FU but not in U87MG cells treated with 25 µM 5-FU. Upon 50 µM 5-FU treatment, elongated cells were seen in both treated U87MG and treated U87-MCSF cells. Scale bar: 50 µm. Reproduced with permission from [69].

Its utility as a tissue tracer was also demonstrated in order to highlight retardation in cell growth as well as other morphological changes. For example, U87-MCSF cells treated with even a low dose of anti-cancer drug 5-fluorouracil (5-FU) were successfully investigated after their staining with MB (Figure 3). It was observed with MB that the elongated, spindle-shaped morphology with lengthy processes was present in U87-MCSF cells with 25 mM 5-FU treatment for 72 h, but not in U87MG cells (Figure 3a). However, elongated morphology was seen in both cells treated with 50 mM 5-FU. Further, cytoskeleton staining of untreated and treated samples of U87MG and U87-MCSF cells was done using anti *β*-actin antibody (Figure 3b). The results obtained reinforce the morphological changes observed in MB staining [69].

Problems that arise in practice must be taken into account, where interference may occur which would transform the benefits of using these compounds into undesirable effects. For example, if an injection of heparin should be administrated before the dye administration, it is worth mentioning that the heparin-induced MB also inhibits the assembly of tau protein into filament, acting on the first and on the fourth repeats of the microtubule-binding domain (MBD), as was observed after in vitro studies [11].

If this dye reaches the lung, it seems to have a protective effect on the in vivo lung perfusion as was demonstrated after an ex vivo study performed in isolated rat lungs. The observed effect shows the attenuation of the mitochondrial oxidative damage and was obtained on a group of rats that were intraperitoneally injected with 2 mg/kg of MB 2 h before surgery. The surgery was followed by 45 min of ischemia and 60 min of reperfusion. Important increases in tidal volume (VT), Cdyn, and PaO_2_ were observed in this group, as well as decreases in raw and histological damages. MB could also reduce the lung ischemia–reperfusion injury (LIRI) and pulmonary edema by decreasing the pulmonary wet/dry ratio and the induced release of lactate dehydrogenase (LDH) in lung tissues. Furthermore, MB concentrations between 0.5 and 2 mM, proved to enhance mitochondrial respiration. After the elucidation of the MB effect on ROS, the treatment markedly reduced, as expected, the production of ROS, the content in mitochondrial membrane potential (MMP) and malondialdehyde (MDA) as well as the ameliorated decrease in superoxide dismutase (SOD) activity induced by LIRI inhibition of mitochondrial swelling induced by ischemia–reperfusion and apoptosis. The increase in ATP production has been also observed together with the other mentioned processes [70]. It was also observed that MB effect depends on its concentration; optimal in vitro effect on mitochondria was obtained with 100 nM of MB solution [71].

### 3.2. Proflavine Uses for In Vitro Studies

In the same manner as in the case of MB, it was also observed that PRO acts in a concentration-dependent manner and is reversible by directly preventing Kir 3.2 activity, sparing the proliferation in normal cells, and hindering the growth of Kir 3.2-expressing cells. The highest magnitude of cell proliferation was obtained at a depolarized potential and after the serial perfusion of 300 mM of PRO solution. This amount of PRO does not affect Kir 4.1 cells, Kir 2.3 cells were weakly affected, and Kir 1.1 cells were moderately blocked, while the current amplitude of Kir 3.2 mutant cells was progressively reduced [60].

When the dye is injected intra-arterial, it has to be taken in consideration that the cationic protonated form of proflavine (PRO+) induces deviation of the simulate structure of human serum albumin (HSA) compared with the crystal structure of proteins without important differences between the bounding sites. A bigger devotion is registered when in its cationic form, where it is forming bonds with BS1. Total binding of HAS simulated structure forms small crystals, with 56% total content of HAS in α-helix, while PRO+ was only half at any of the considered binding sites, in BS2. The circular dichroism measurements show that PRO+ did not impart any substantial changes to the secondary structure of HAS [59].

The relatively easy penetration of PRO in the epidermis cells make it a good skin tracer but also an unharmful compound for viral tropism. In vitro studies performed to date have demonstrated that PRO bound irreversibly to the herpes virus, and after removal of unbound dye, the rapid inactivation of the virus could be accomplished by brief exposure to fluorescent light. It was observed that the dye molecules are localized in some specific sites of the herpes simplex viral replication like in cells of the epidermis, where the penetration of the epidermis occurs relatively easier compared with other sites [72].

It was reported that human fibroblasts or primary bronchial epithelial cells treated with PRO were partially protected against Semliki forest virus or rhinovirus infections induced after more than 24 h of contact with anti-viral genes. PRO presents its inhibitory effects on topoisomerase I and II and DNA recombinase-toxicity activating the repairing mechanism of DNA after acriflavine intercalation and cytoplasmic leakage of DNA products. PRO, acting indirectly on cGAS-cGAMP-STING axis on human and murine cells, leads to DNA leakage into the cytoplasm. Its effect on DNA plays an important role in the overall antiviral activity. The response obtained when sub-toxic doses of PRO were used, was directly dependent on cell type, since cytoplasmatic DNA leakage was absent in L929 murine cell, which express functional levels of cGAS and STING. However, the DNA damage was still present [62].

**Figure 4:**
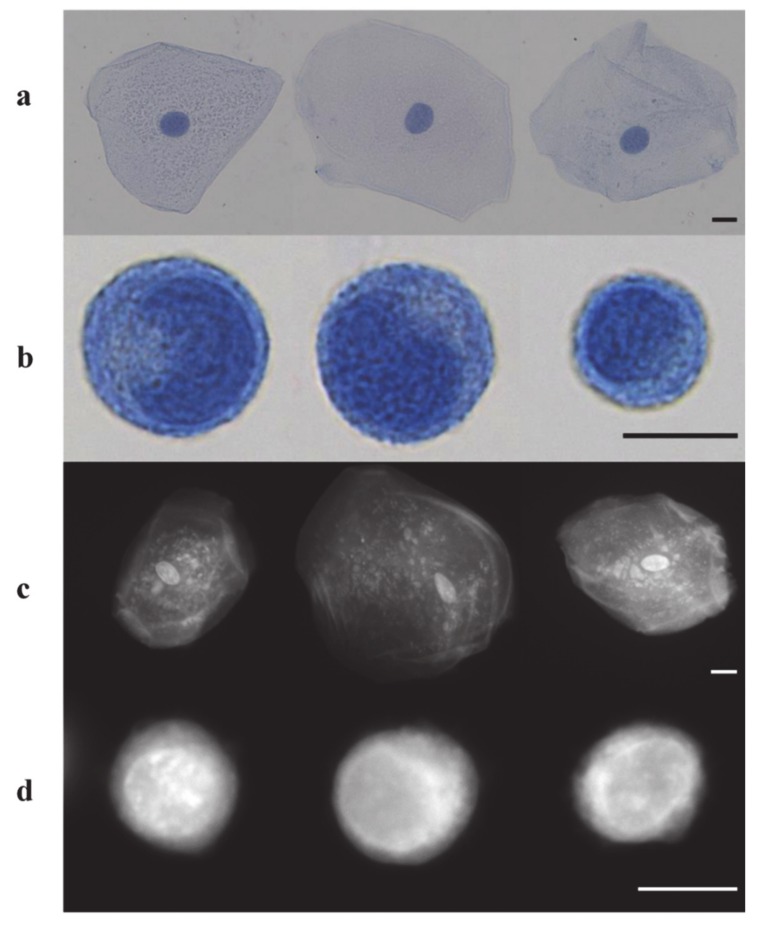
Visual diagnosis comparison of Papanicolaou and proflavine-stained normal oral cells and CAL 27 cell line. (**a**) Papanicolaou-stained normal oral cells. (**b**) Papanicolaou-stained CAL 27 cells. (**c**) Fluorescent images of proflavine-stained normal oral cells. (**d**) Fluorescent images of proflavine-stained CAL 27 cells. Scale bars = 10 μm. Reproduced from [73] (open access article).

PRO’s ability as a tissue tracer, which does not require an incubation period, is sustained by its applicability as a rapid stain for cytologic examination of biological specimens since it fluorescently stains cell nuclei and cytoplasmic structures. This important characteristic is due to its small amphipathic structure and ability to intercalate DNA. PRO was used as a rapid cytologic dye on a number of specimens, including normal exfoliated oral squamous cells, cultured human oral squamous carcinoma cells, and leukocytes derived from whole blood specimens using a fluorescence microscope. No incubation time was needed after suspending cells in 0.01% (*w*/*v*) PRO prepared in saline medium. Images of PRO stained oral cells had clearly visible nuclei as well as granular cytoplasm, while stained leukocytes exhibited bright nuclei, and highlighted the multilobar nature of nuclei in neutrophils. The utility of digital images of PRO-stained cells, which can be used to detect any morphological differences between different cell types, was also demonstrated. It can be noticed that Giemsa stains both erythrocytes and leukocytes (Figure 4a,b), while PRO preferentially stains the nuclei-containing leukocytes (Figure 4c). This phenomenon is due to the fact that human erythrocytes have low DNA content, thus it determined a low fluorescence signal from these cells. While the erythrocytes are not visible with PRO staining, the monolobar and multilobar structures within leukocytes are clearly distinguishable [73]. 

## 4. In Vivo Studies

In vivo studies performed on animal models and clinical studies not only demonstrate the lack of toxicity but also prove the benefits of the use of MB, to act not only as a dye but also as a substance that determines the improvement of the tissue state.

### 4.1. The Use of Methylene Blue on Animals 

The scientific and practical value of the results obtained from in vitro tests is well recognized, but to apply the results in medicine it is necessary to move the research to another level, this being constituted by studies on live animals. Although the majority of in vivo studies performed to date were made in order to study its effect on many pathologies and other proprieties than the ones well-known, they also revealed MB’s action on different tissues. Whether those actions take place depends on the concentration and tissue reached by the dye when is injected intra-arterially, if mapping of skin vascularization is intended.

The first example is related to the effect of long-term treatment of mice suffering from Leigh syndrome. It was observed that treatment with 0.125 µM (125 nM) MB solution showed important increases in the metabolic activity of the treated mice, while the diet which included 27 mg MB/kg administrated from 4 months of age to UM-HET3 mice, improved female lifespan by 6% [74]. Despite this, there are studies on long-term administration of MB (24 months) in rodents which revealed the formation of lymphomas and the activation of various intestinal malignancies [75].

Administration of MB doses of 74–208 ng/kg not only protected the mice from endotoxic shock but also activated an antidepressant-like response. In rats, the same doses improved cognitive function while the administration of a single, but higher dose, increased the activity of cytochrome c oxidase (complex IV) in the brain by about 25% [74].

Depending on concentration, MB can even penetrate into the central nervous system. The treatment of rat brain with 1 mg/kg MB intraperitoneally administrated for 15–30 min after spontaneous intracerebral hemorrhage, with 0.5 mg/kg administrated after 6 h and 1 mg/kg administrated daily for the next 3 days led to the following effects:

- Anti-apoptosis as a result of the PI3-Akt activation and GSK3β inhibition in the peri-hematoma brain tissue;
- reduction of the brain tissue edema in ipsilateral basal ganglia;
- the increase of the myeloperoxidase (MPO) number and Iba1 positive after the first 24 h;
- reduction of the pro-inflammatory cytokines (e.g., interleukin (IL)-1β, tumor necrosis factor (TNF)-α, and IL-6) effect and upregulation of IL-10 cytokine that exerts anti-inflammatory and neuroprotective effects;
- reduction of the neutrophil infiltration and microglia activation.

Akt is a member of the serine/threonine kinase family which acts in cell survival, proliferation, and apoptosis. Acting in PI3K/Akt/GSK3β pathway, it plays an important role in the anti-inflammation effect of MB after intracerebral hemorrhage. MB treatment did play a positive role in the reduction of the intracerebral hemorrhage (ICH) effect. Wort, a selective inhibitor of PI3K reverses antiapoptotic and anti-inflammatory effects of MB [76].

Brain oxidative lesions indicators show differences in piglets when 3 mg/kg MB was administrated at the time of, and 60 min after, CRP associated with delayed hypothermia (33–34 °C), if compared with standard resuscitation; when comparing postponed hypothermia with standard CRP no difference was observed. Except for lucigenin, all indicators registered important differences in the group with postponed hypothermia and MB, compared to the one without MB. The first group had the best beneficial results in the case of both survival and extent of cerebral neuropathological injury. It was also observed that postponement of hypothermia by 30 min in cardiac arrest was compensated by the administration of MB [77].

Several benefits for the central nervous system were also reported as consequences of MB in vivo administration, namely, the improvement of cognitive function and increased activity of cytochrome c oxidase (complex IV) in the brain after a single high dose administrated in rats; improvement in the lifespan of UM-HET3 female mice after 27 mg MB/kg regular diet applied to subjects at 4 months; increase of the metabolic activity in Leigh French Canadian variant suffering of Leigh syndrome after treatment with 0.125 µM (125 nM) MB; protection of mice from endotoxic shock as well as eliciting an antidepressant-like response after the administration of 74–208 ng/kg MB [74].

The hermetic dose-responses were observed after i.v. administration of the dye via the tail vein. Amounts of MB between 1.87 and 60 mg/kg were used for adult male Wistar rats, with important anxiolytic effects observed in the range of 7.5 to 30 mg/kg while antidepressant-like effects appeared at doses of 15 and 30 mg/kg. If MB is intraperitoneal (i.p.) administrated, it induces an anxiolytic effect at doses of 1 mg/kg, showing the prominent role of the nitric oxide-guanosine monophosphate (NO-cGMP) pathway in the genesis of anxiety [78].

MB can influence the vascular caliber by acting on NOS, an important enzyme in vascular tonus. In the case of experimental studies performed on animals, the control of bleeding led to a progressive decrease of blood pressure in response to the action of vasoactive substances. The blood transfusion will not restore the initial pressure, the effect being reversed by the pharmacological inhibition of nitric oxide (NO) synthesis. Administration of MB with Ringer lactate increases the survival rate, arterial pressure, cardiac output, vital end-organ blood flow, oxygen delivery, and decreased serum lactate levels in canine models compared with MB or Ringer lactate alone. In rats resuscitated with fluid which received an early injection of MB, an improvement of survival rate was registered in the case of controlled and uncontrolled hemorrhages. In the case of hemorrhagic shock MB reduces the volumes of shed blood and fluids required [79].

The inhibitory MB concentration for NOS in the case of the intracerebral administration on rats was 2.7 µM. In addition, 10 mg/kg p.o. lead to a mean maximal concentration in plasma of 86 ng/mL (0.27 µM), which determines a MB concentration of 0.27 µM in the brain, making interaction with **monoamine oxidase B (**MAO-B) unlikely for in vivo tests. When MB was orally administered in drinking water to old mice for three months, it determined a brain concentration which does not exceed 120 nM [71]. Although after 1 h, the brain concentration is still 10–20 times higher than in the circulatory system, indicating a rapid and extensive accumulation of MB in the nervous system [11]. If 1 mg/kg of MB is intraperitoneally administrated, it results in 0.5 µM of dye concentration in the brain. The antidepressant effect of MB administration on adult male Wistar rat manifests in a U-shaped dose-response manner, only at higher dosage amounts of 15 to 30 mg/kg for intraperitoneal administration and for lower dosage amounts of 10, 30, and 100 nmol/µL for administration in the dorsal periaqueductal gray area (dPAG) via microinjection. If higher concentrations of MB solution are used, dimers are formed that are toxic for cells; this is probably due to their accumulation in the cytoplasmic organelles of cells [63].

Like for in vitro studies, when the effect of MB varies with cell type, the in vivo effect varies both with dose and animal species. For example, MB has an exceedingly high volume distribution of 21.0 L/kg in rabbits, while in rats, MB speeds up ethanol elimination increasing the metabolism of ethanol to CO_2_ [11]. In a rabbit model, low MB doses between 0.025 and 3 mg/kg were used to color both thyroid and parathyroid glands for imaging diagnostics [80].

When the intravascular injection is considered as the route of administration, the optimal concentration is determined starting from the value of the half-lethal dose (LD)50 of MB, that is, 1180 mg/kg in rats and 3500 mg/kg in mice if orally administrated. The intraperitoneal administration of MB determines the dramatic decrease of the LD50 of MB at 150 mg/kg in mice and 180 mg/kg in rats, respectively. The i.v. administration of MB determines a large variability in LD50 values as follows: 77 mg/kg in mice, 1250 mg/kg in rats, and 42.3 mg/kg in sheep. IC50 values ranging from 27 to 180 nM of MB inhibit selectively the function of MAO type A [11], while an IC50 value of 0.51 µM has dual target antidepressants after the administration of a single MB dose of 0.07 µM [63].

Butyrylcholinesterase (BuChE) is inhibited in a concentration-dependent manner, with IC50 values ranging between 0.4 μM and 5.3 μM, but this effect differs in the presence of an isoenzyme such as AChE versus BuChE, and it varies in a species-dependent manner. In guinea pig liver homogenates, 0.5 μM of MB inhibits the activity of MAO, potentiates the vasoconstriction induced by tyramine, and forms bonds with MAO in mouse brain. *L*-arginine induces the release of dopamine in the striatum and increases the extracellular levels of serotonin and dopamine in the medial preoptic area [11].

By knowing the metabolization rate of MB and also its concentration in tissue for different ways of administration, the optimal concentration for vascular mapping can be estimated.

One possibility to avoid flap necrosis and poor wound healing, without using subjective measurements, is the use of intraoperative assessment of perfusion, thus avoiding rising areas with insufficient amounts of the perfused substance. MB has been found to be suitable as a tissue tracer because it is long lasting in this environment, thus able to provide a visual map of tissue perfusion. The necrosis prediction accuracy of MB injection was estimated at 91.9%, the sensitivity was 91.8%, and the specificity was 92%, respectively, at about 5 min after administration. Despite all these advantages of i.v. administration of MB, in clinical practice the administration of bolus of selected but lower concentrations was preferred in order to avoid high peak concentrations and to eliminate possible side effects [81].

There are already some specialized studies which evaluate the suitability of MB for vascular mapping and evaluation of vascular permeability. MB suitability in coronarography was tested and it was observed that if the dye is injected intracoronary it will be extracted by the myocardium, then used for the visualization of miocardic perfusion and arterial flow. An MB bolus was i.v. administrated and its fluorescence peak was revealed at 700 nm after 12 s. This signal shows persistence for more than 10 min and returns to normal after 40 to 60 min. Intracellular metabolization of MB permits direct visualization of heart ischemia via the oxidative status. In this case MB is reduced to leucomethylene blue (see the chemical formulas of this compound in Figure 2) which is colorless, the florescence pick is reduced, the signal rapidly returns at its base line, and the environment has a highly oxidative status. As proven, the MB concentration, excitation rate, and duration of camera exposure critically influence the fluorescence intensity and varying these parameters can be modulated to improve the analytical signal. For example, the MB dose should be reduced by 50% by doubling the excitation rate or the time of the camera exposure, or by 75% by doubling both other parameters [82]. A solution of 1% MB prepared in water was used at a dose of 2 mg/kg and 1 mg/kg, respectively. Boluses of this solution were i.v. administrated in 30 s on 300 mg rats with 25 mg of Indocyanine Green ICG in a dose of 0.06 mg/kg for near-infrared (NIR) fluorescent imaging applying a wavelength of 700 mm. The absorption and emissions bands of MB are at 667 nm and 686 nm, respectively and the correspondent coefficient of extinction was found to be 46,000 M^−1^ cm^−1^ which indicates low-to-moderate total fluorescence if compared with other NIR fluorophores. It was observed that the MB administrated i.v. does not bind to proteins. Thus, 2 mg/kg of MB was i.v. injected in rat and revealed a high uptake of dye in the myocardium with a peak that appears after 28 s. If the MB was administrated in other organs, the peak appeared between 15 and 57 s after injection and slow washout, with the NIR fluorescence signal returning back to ground levels after 40 to 60 min, depending on the organ type. The last example was registered for the kidney. The kidney and liver are organs which excrete MB, thus permitting intraoperative mapping of the bile ducts and ureters [82].

NIR tests were also performed on pigs. Here only a small volume of 10 mL of saline 31.3 mM MB solution which is the equivalent of 2 mg/kg was needed for a single NIR florescence image taken at 5 min post injection and 500 ms camera exposure time. The fluorescence signal was then slowly decreased reaching the baseline after 1 h. During the arterial phase, MB is extracted by skin being a relatively stable intracellular florescence [83]. MB has found applications as a true perfusion tracer, since it provides sharp borders between perfused and under-perfused areas. This information could be obtained by registering a single sub-second high-resolution image immediately after flap creation; this strategy generates high accuracy in predicting flap perfusion [83]. Other studies present the possibility of using MB to highlight the split in central retinal artery or stain the sclera without side effects. These tests were done with good results on rabbits, by injecting 10 mL of MB solution at a flow rate of 0.5 mL/s in facial artery [84]. This principle was also exploited in another study where rats’ lymph nodules where investigated after the injection of 0.1 mL of 1% MB solution which allowed the efficient study of flap viability [85]. The intra and perioral injection of small volumes that do not exceed 20 µL of 0.25 mg/mL MB solution showed similar florescence and affinity in skin and muscle, good tolerance, and fast metabolization [81]. It should also be mentioned that if a flap will rise based on the vascular map obtained by MB injection, it will experience an ischemia–reperfusion phenomenon.

Clean-grade C57/BL6 mice aged 4 months were continuously treated with gavage of MB solution of 25 mg/kg/day until they were 7 months old. Bilateral renal pedicles were carefully separated and occluded using a micro-artery clamp that was removed only after 45 min and the kidney turned red rapidly. It was observed that MB limits the renal dysfunction in IR, according to the low levels of serum creatinine (SCr) and blood urea nitrogen and attenuates pathological changes in renal tissue on rat. It also downregulates IL-1β and IL-18 and upregulate IL-10 and TGF-β1. Thus, it can be noticed that pro-inflammatory factors are inhibited by MB, and at the same time, anti-inflammatory factors are favored; these processes result in the attenuation of the inflammatory response. In the same study it was demonstrated that MB downregulates ROS, NFB, and NLRP_3_ by the antioxidant effect which reduces NFkB and NLRP_3_ proteins expression by ROS/NF-KB-NLRP3 pathway. Furthermore, caspase-1 pathway is downregulated by MB. Caspase-1 mediates apoptosis and promotes the maturation of inflammatory factor IL-1β although pro 1L-1β expression is high, alleviating renal tissue lesions and inflammatory response in brain trauma in mice [10].

In the case of 4 h-old hind limb ischemia, the intraperitoneal administration of 2 mL of 1% MB (50 mg/kg) and its 10 min reperfusion before or after ischemia-reperfusion, it was observed that the use of MB attenuates skeletal muscle damage. The histological damage score in lung IR lesions by reducing tissue edema, neutrophilic infiltration, THF-α, IL-1β, IL-6 levels, decreases the MPO activity and MDA level, and increases SOD activity in lung tissue. MB also permits hypoxanthine and xanthine oxidation in uric acid preventing superoxide formation as an alternative co-substrate for oxidation. The treatment with MB before IR has the same results as after IR [54].

If a flap is preconditioned with a combination of norepinephrine and MB, the overall vascular resistivity after norepinephrine infusion is dose-dependent, vascular resistance rises dramatically, and at 10^−5^ M the basic maximal constriction is 520% and EC_50_ is 3.18 × 10^−6^ M. A single pedicle of the isolated perfused porcine skin flap (IPPSF), axially patterned, and tubed, responded to MB concentrations from 10^−4^ M to 10^−3^ M and 10^−5^ M norepinephrine. Continuous infusion of the same volume produces a sustainable response compared with bolus administration. When the infusion is stopped, the vascular resistance drops immediately and, in a few hours, gradually returns to the basal level. In this case, concentrations of 10^−3^ M and 10^−8^ M of acetylcholine cannot sustain the drop of the vascular resistance. Under the same conditions, the nitroglycerin concentrations from 5 × 10^−8^ M to 5 × 10^−6^ M seem to be inefficient in lowering vascular resistance but a concentration of 5 × 10^−5^ M suddenly drops the vascular resistance. The effect is sustained only at the time of infusion, after that it dramatically rises. At norepinephrine concentrations from 10^−5^ to 10^−3^ M and higher amounts of acetylcholine, the glucose consumption is reduced constantly, with maximal effect of 25% under base line, this being in contrast with nitroglycerin and tolazoline influence which constantly increase the glucose utilization with maximal response at 5 × 10^−5^ M. The effect of acetylcholine is more efficient in the presence of MB than nitroglycerin, suggesting the direct action of MB on EDRF, which first has to cross the extracellular space, then acts on smooth muscles [86].

One of many experimental uses of stain on the skin is in Mohs micrographic surgery, a procedure that permits the identification and removal of tumor at the surgical margin. In this case, an ex vivo, horizontal sectioning technique is used in order to map the tumor boundaries. This particular procedure yielded a remarkably low recurrence rate. However, this procedure is time-consuming and costly, since it requires a specially trained surgeon and a dedicated laboratory and technician for processing high-quality frozen sections. The development of in vivo imaging techniques able to assist the surgeon in identifying tumor margins, both preoperatively and intraoperatively, could therefore be very valuable.

The in vivo action of the dye on skin, tested on rats, showed that MB applied on the skin from outside, disappears between a few hours and 12 days but not beyond 2 weeks [87]. Unlike this, the MB injected intraperitoneally acts in total thickness burn lesions, reducing necrosis rate inversely proportional with the time passed from the lesion until MB was administrated. Thus, a 44.7% or 13.3% decrease of necrosis was obtained by reducing cell damage due to oxidative stress, if 2 mg/kg of MB was administrated intraperitoneally on rats at 1 h and 6 h after the lesion production, respectively. Higher MB doses from 65 to 200 mg/kg administrated i.p. were used in order to evidence and to study the side effects of this treatment. In the areas with inflammation and ischemia, but without necrosis, the percentage of reepithelization is greater for the groups treated histologically, while the percent of normal tegument without inflammation and ischemia is greater among the rats treated with MB. The histological finding were successfully correlated with the photographic assessments [88].

The kinetic of this dye can be assessed knowing that 3 mL of MB, with a concentration of 10 mg/mL, was injected with a flow rate of 3 µL/s in female mice kidneys to assess the urinary flow in mice embryos ureter until it reaches the bladder. It was observed that it takes about 5 s for dye to reach the bladder if the ureter has a proper insertion. This volume of 3 mL provided the dye flow for 20 s after injection in the kidney [89].

**Figure 5:**
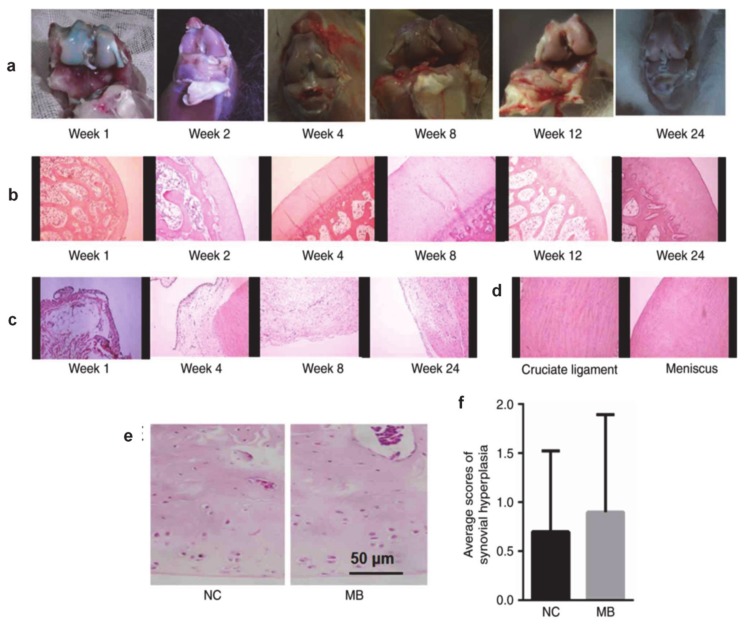
Intra-articular injection of MB is safe in rabbits. (**a**) No notable morphological changes were observed in rabbit articular cartilage at 1, 2, 4, 8, 12, and 24 weeks following MB injection. (**b**) The rabbit cartilage was intact, and the chondrocytes and matrix were normal at all time points, as indicated by hematoxylin and eosin staining at 100× magnification. (**c**) No synovial hyperplasia was observed at 1, 4, 8, or 24 weeks following injection of 1 mg/kg MB into the rabbit knee joint cavities at 100× magnification. (**d**) No lesions of the cruciate ligament or meniscus were observed 24 weeks following injection of 1 mg/kg MB into the rabbit knee joint cavities at 100× magnification. (**e**) No histological characteristics of articular cartilage sections were observed in the MB or control groups 24 weeks following the injection of 1 mg/kg MB. (**f**) There were no significant differences between the synovial hyperplasia scores of the control group and the MB group. MB, methylene blue; NC, normal control. Reprinted from [90] (open access reprint conditions).

In the case of the peripheral nervous system, MB proved to be an effective tool applied for the treatment of pain and inflammation induced by osteoarthritis. Thus, doses of 1 mg/kg were administrated in rabbits as a local injection of 0.2% MB solution. Due to the high affinity of MB for the nervous system, administration of this dye suppresses long-term peripheral nerve medulla pain and reduces the levels of inflammatory factors (e.g., IL-6, TNFα, IL-1β, and IL-8) without relevant histological intra-articular damage. The results obtained from this study are presented in Figure 5 [90]. 

There was another study which also reported the use of MB as an intraoperative stain, without excising the stained tissue. In this case, it was proven that the absence of collateral tissue necrosis and architectural disturbances was intraoperatively stained for the grafts used in tympanoplasty. Thus, 300 µg/mL of MB solution was intra-tympanically injected into guinea pigs and it shows a pure-tone audiogram. This test revealed important and successful improvement in hearing for all tested [91].

MB’s florescence property to stain endothelial cells, which can be used to map the skin vascular system in a specific area, was used in gliomas to differentiate between tumoral cells, vessels, and normal tissues based on absorption band value. These studies used 1% MB solution injected directly into the gliomas [92]. Furthermore, only 35 μL of 500 μg/mL MB solution that was in vivo injected intratumorally in the Cremophor vehicle, showed a greater degree of homogeneity for MB fluorescence tested in water. If the tumor is irradiated, MB fluorescence restores progressively, even exceeding the pre-irradiation levels compared with non-irradiated levels which decrease constantly. This concentration of MB shows statistically important EMT6 tumor growth delay [93].

It can be stated that only a few examples of the in vivo use of MB have been listed above, many other applications are presented in the literature. Thus, if a simple search is done via the ScienceDirect database directly with the keywords, “methylene blue” and “biomedical applications”, 6275 research papers and review-type articles are found. Furthermore, by using “methylene blue” and “clinical applications”, 12,470 research papers and review-type articles are found. 

### 4.2. The Use of Proflavine on Animals

PRO, like MB, is an efficient tissue tracer, but with a more rapid transfer to tissue if administrated both i.v. and intramuscularly (i.m.) [94].

The lower limit of quantification for PRO was determined to be 0.05 mg/mL in plasma, urine, and liver samples, while the LD50 was found to be 0.33 mg/kg in the case of i.v. administration, 1.67 mg/kg in the case of bolus administration, and from 1 to 5 mg/kg in the case of intramuscular (i.m.) administration. These findings were very important to determine the concentration needed for intra-arterial injection to map skin vascularization [95].

Several biomedical studies performed on PRO to date have allowed the evaluation of PRO activity on living tissues. One such example was the use of PRO as an antineoplastic agent, in which PRO concentrations of 1.67 mg/kg or 1.87 mg/kg were considered for i.v. administration while 5 mg/kg of dye was applied for i.m. administration. When i.v. injected, the PRO concentration decreases rapidly in plasma within the first 3 min followed by a slower decrease with a half-life in plasma of 0.568 min when 1.67 mg/kg of PRO is administrated, while for a dose of 1.87 mg/kg the half-time is 0.66 min. Next, a slower decrease in concentration was registered for 6.54 min in the first case and for 6.60 min in the second case, respectively. When i.m. injected, PRO has about a five-fold higher half-life, meaning 41 min, and 3.6% cumulative excretion in urine from the quantity administrated after 24 h, as compared with 2.1% in the case of i.v. injection. Due to its high bioavailability that reaches 85%, as well as their tissue affinity, PRO is an excellent micro-vascular tracer, similar to MB. PRO presents a relatively stable tissue concentration after 60 min from the administration, then its concentration continuously decreases, reaching below the noticeable limit after 48 h [94]. PRO can be found in all tissues since it presents rapid tissular distribution, thus, high levels were found in kidney, even greater than C_max_ which is 1.71 mg/mL [95]. In the case of i.m. administration, the association with guanosine prolongs the presence of PRO in plasma and reduces the biliary and urinary excretion by about 1.25-fold after i.v. administration of the bolus and 2.1-fold after i.m., prolonging injection together with the 10-fold increase of bioavailability [94,95].

PRO is fully excreted in the bile 5–6 h from its administration, but the cumulative amount excreted in bile after 12 h was calculated to be 14.8% from the quantity administrated in the case of i.v. injection, and 4.8% in the case of i.m administration. The amount excreted in urine after 24 h from the administration was calculated to be about 2.6% in the case of i.v. injection and 4.6% in the case of i.m. administration. The animals used for the in vivo studies tolerated maximum amounts of PRO at 10 mg/kg well, with no adverse effects reported [95].

If combined with in vivo microscopy, the injection of PRO in tissue allows the assessment of precancerous lesions [35]. Thus, only 1 mL of 0.01% PRO solution topically applied and associated with digital photography helped the surgeons to determine the most suitable places for performing biopsies [36].

Although PRO preferably stains cell nuclei, it can also be used to evaluate crypt morphology by topical staining the colorectal mucosa. The hemisulfate form of PRO was used to stain porcine and caprine bulk colorectal tissue by using solutions of 0.01%, 0.05%, and 0.10% prepared in phosphate-buffered saline. By increasing the concentration of PRO, the signal intensity increases [9]. It was observed that doses of 0.01% PRO i.p. injected in mouse and human cells increases antiviral immunity via the STING mechanism. If this amount of dye was applied for 3 successive days, the viral titers drop about 100-fold [35].

Compared to MB, PRO has less applications on animals, but the results obtained show that PRO is a promising candidate for multiples uses in clinical practice. In the case of PRO, the statistical study revealed a much small number of 471 research papers and review-type articles when using the terms “proflavine” and “clinical applications”, while by using the terms “proflavine” and “biomedical applications” only 292 papers were found.

### 4.3. Clinical Trials of Methylene Blue and Proflavine

The encouraging results obtained for both MB and PRO after in vitro and in vivo tests, as well as the promising results regarding their toxicity have led researchers to include these compounds in clinical trials for different applications, such as their role as tissue tracers, early-stage diagnostics of different cancers, and many more; some of which were discussed in this study.

**Figure 6:**
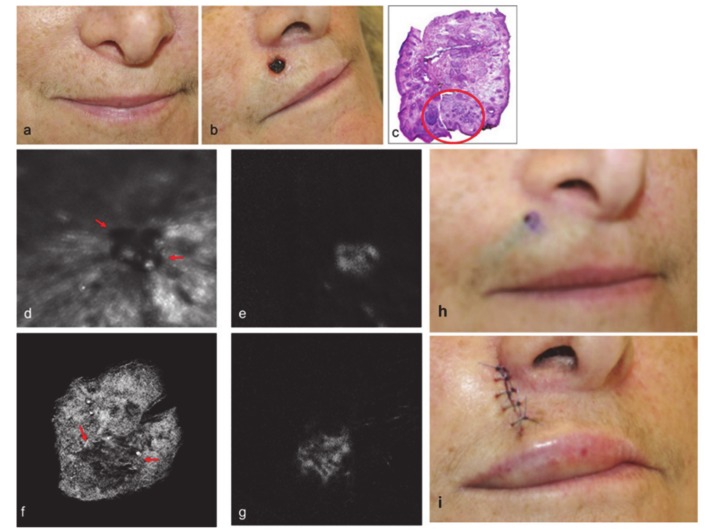
Clinical images of a 63-year-old female patient diagnosed with infiltrative basal cell carcinoma BCC. (**a**) Clinical preoperative picture. (**b**) Clinical postoperative picture. (**c**) Hematoxylin and eosin (H&E) section of the excision of first Mohs stage with a positive margin (tumor outlined by red circle). (**d**) In vivo reflectance image at 630 nm after the first Mohs stage (field of view (FOV) = 14.2 mm). (**e**) In vivo fluorescence polarization image after the first Mohs stage (FOV = 14.2 mm). (**f**) Ex vivo reflectance polarization image of the stage I excision (FOV = 12 mm). (**g**) Ex vivo fluorescence polarization image of the stage I excision (FOV = 12 mm). Red arrow shows the tumor. (**h**) Preoperative picture after MB injection. (**i**) Postoperative picture after repair. Reprinted with permission from John Wiley and Sons [96].

Multi-spectral polarized light imaging and MB fluorescence polarization imaging have been reported as methods for real-time, intraoperative delineation of non-melanoma skin tumor margins, after some ex vivo tests. In this proof of concept study, a novel technique that integrates polarization-enhanced reflectance and fluorescence imaging (PERFI) for its potential to assist surgeons in demarcating nonmelanoma skin cancer NMSC margins in vivo during Mohs micrographic surgery was described. In this study it was demonstrated that the injection of MB was well tolerated, a transient blue staining within the treated area was first observed, which disappeared completely within 1 week in all of the patients considered for this study. The ex vivo polarization esulting images (PLI) and fluorescence resulting images (FPI) of the excision correlated well with histopathology images, as observed in Figure 6 [96]. The limitation in the case presented here was that the marked skin was excised during the operative procedure.

Few clinical tests on human subjects have been performed to date in order to reveal the possibility to use MB in oral precancerous and cancerous lesions detection. All these clinical tests were evaluated based on the estimation of some analytical parameters, such as sensitivity (low number of false negatives), specificity (low number of false positives), and predictive values. For example, Riaz and coworkers performed a clinical study in which they enrolled 100 patients (50 cancerous, 50 precancerous) and 20 healthy subjects as controls and used MB as the stain agent for oral lesions. An average sensitivity of 91.4% was obtained with only 8.6% false negative results, making this study very promising for using MB dye for early diagnostic screening since all the clinical suspicions of malignancy were proved pathologically after biopsy. If compared with control, 0 false positive results were obtained, thus the specificity was 100% and the diagnostic accuracy was 90% (*p*-value < 0.001) [40].

Detection of early cancers or precancerous lesions in the stomach was also envisaged as an important application of MB in clinical trials. The low validity and reliability of endoscopic techniques for the detection of subtle histological changes as well as determination of the extent of histological abnormalities required to plan a surgical resection or endoscopic ablation therapy are envisaged. Thus, staining the gastric mucosa with MB was often applied for the identification of areas of nonacid-producing mucosa, but in this study, it has proven to be useful for the detection of at least one of the premalignant gastric lesions, namely intestinal metaplasia. Only patients with previous positive biopsy results were included in the study, thus the specificity and the sensitivity of the procedure cannot be considered but should be regarded as an approximation; further studies including human subjects without a previous diagnosis of metaplastic lesions are needed for this purpose [41]. However, the results proved encouraging for the screening of early-stage cancer of the stomach.

In the case of PRO, this dye was successfully applied in high-resolution microendoscopy for in vivo diagnostic of cervical epithelial cells. The risk of cervical disease progression was followed for 232 patients that were exposed to PRO and 160 patients without exposure to the dye (control). All the patients underwent treatment and follow-up based on histopathologic results and per the local standards of care for 18.7 and 20.1 months. Progression of disease was evaluated by comparing histopathology from the initial tests to the worst registered one during the monitoring period. It was observed that there were no noticeable differences in disease progression from the initial diagnostic to invasive cancer between the proflavine exposed and control groups overall. Some risks of cervical dysplasia progression were observed, but these findings were in good correlation with those observed for the natural cases of cervical cancer [35]. These findings are thus promising for using PRO in early diagnostic.

## 5. Conclusions

Used at the beginning only for their antiseptic and respective antiviral activity, MB and PRO have turned out to be excellent dyes for diagnostic and treatment of methemoglobinemia, septic shock, and ifosfamide-induced encephalopathy.

The in vitro studies performed to date proved that MB has several benefits such as the delay of senescence for fibroblasts, the mediation of the photo modulation on human osteoblasts, the increase protection against irradiation, brain damage, poisoning, chemotherapy, as well as the enhancement of mitochondrial respiration. These benefits are all correlated with the anti-inflammatory and antioxidant effects of MB which are also dose-dependent, thus it is very important to choose the right amount of MB for each application. In vivo studies also demonstrated that MB improves cognitive function, antidepressant effects, and reduces brain edema in intracerebral hemorrhage, in ischemia-reperfusion syndrome as well as the necrosis rate in total thickness burn lesions. Thus, the administration of MB proved to be an effective method for the treatment of pain and inflammations induced by osteoarthritis.

Many of these effects made MB an excellent dye to map the vascular network of the skin, like in the case of perforasome, but also allowed the assessment of its perfusion with benefic effects on the tissue. Like in the case of MB, the effects of PRO were found to be strictly correlated with its concentration and also with the route used for the administration.

In this review the main applications of MB and PRO were revised in a critical manner, insisting on the positive results obtained in the case of in vitro, in vivo, and clinical tests performed on humans. Although MB has been known and used in medical applications for several decades, new assignments are foreseen especially in assisting reparatory surgery. Due to their optical properties both compounds can be spotted by the naked eye (even in relatively low concentrations) or by using optical techniques (fluorescence or NIR).

It can thus be concluded that by hyphenating the traditional surgical methods with new analytical devices, procedures or the use of different compounds with special properties will lead to the improvement of surgical techniques with a direct and beneficial impact on human health.

## Figures and Tables

**Table 1:** Methylene blue in clinical uses.

Application	Mechanism/Explanations	Dose	Ref.
Preoperative and intraoperative use in cardiac surgery	MB has been used intravenously (i.v.) for over 30 min in patient’s in the intensive care unit (ICU) 1 h before surgery and a decreased incidence and severity of vasoplegic syndrome in high-risk patients was found.	MB solution (1%)	[25]
Septic shock	MB is an inhibitor of guanylate cyclase, very effective in improving the arterial pressure and cardiac function in septic shock.	Not reported	[26]
Hepatopulmonary syndrome	MB is found to increase arterial O_2_ pressure and to decrease alveolar-arterial difference for partial pressure of oxygen in all patients with hepatopulmonary syndrome. MB is a potent inhibitor of guanylate cyclase.	Not reported	[27]
Antimalarial	MB is an effective and cheap antimalarial agent especially in countries with increasing resistance of *Plasmodium falciparum* to existing first line antimalarial agents.	Dose of 36–72 mg/kg MB over 3 days is the most effective scheme of treatment	[20,21,22,23,24,28]
Methemoglobinemia	MB acts by reacting within red blood cells (RBCs) to form leucomethylene blue, which is a reducing agent of oxidized hemoglobin converting the ferric ion (Fe^3+^) back to its oxygen carrying ferrous state (Fe^2+^).	Dose of 1–2 mg/kg of 1% MB solution	[29]
Ifosfamide neurotoxicity	MB acts as an alternative electron acceptor and reverses the nicotinamide adenine dinucleotide NADH inhibition of hepatic gluconeogenesis while also inhibiting the transformation of chloroethylamine into chloroacetaldehyde. It also inhibits multiple amine oxidase activities, preventing the formation of chloroacetaldehyde.	Not reported	[30]
In cancer	MB and other redox cyclers induce selective cancer cell apoptosis by nicotinamide adenine dinucleotide phosphate NAD(P)H: quinine oxidoreductase (NQO1)-dependent bioreductive generation of cellular oxidative stress. MB is also investigated for the photodynamic treatment of cancer.	Not reported	[31]
As dye and stain	MB is used as a safe and effective method of localizing abnormal parathyroid glands, for intraoperative endoscopic marking of intestinal lumen and for location of different lesions.	Concentration from 0.05 μM to 1 M of MB solutions	[32,33]
